# Pan-Cancer Analysis of OLFML2B Expression and Its Association With Prognosis and Immune Infiltration

**DOI:** 10.3389/fgene.2022.882794

**Published:** 2022-07-06

**Authors:** Pengbo Hu, Xiuyuan Zhang, Yiming Li, Liang Xu, Hong Qiu

**Affiliations:** Department of Oncology, Tongji Hospital, Tongji Medical College, Huazhong University of Science and Technology, Wuhan, China

**Keywords:** OLFML2B, pan-cancer, prognosis, TME, immune infiltration

## Abstract

**Background:** The function of olfactomedin-like 2B (OLFML2B), as a member of the olfactomedin domain-containing protein family, remains ambiguous, especially in tumors. The current study explores the possible correlation between OLFML2B, prognosis, and immune infiltration in pan-cancer.

**Methods:** We applied a number of bioinformatics techniques to probe the prospective function of OLFML2B, consisting of its association with prognosis, clinicopathology, alteration, GSEA, tumor microenvironment (TME), immune-associated genes, immune infiltration, tumor mutational burden (TMB), microsatellite instability (MSI), and drug sensitivity in several cancer types. qPCR and immunohistochemistry were used to identify OLFML2B expression in LIHC cell lines and liver cancer tissues.

**Results:** We discovered that OLFML2B was overexpressed in 14 cancers and positively related to several cancer type prognoses. The expression of OLFML2B was further validated in the LIHC cell lines. OLFML2B expression was bound up with TMB in 13 cancers, MSI in 10 cancers, and TME in almost all cancers. Furthermore, OLFML2B was highly co-expressed with genes encoding immune activators and immune suppressors. We further found that OLFML2B played a role in infiltrating different types of immune cells, such as macrophages and cancer-associated fibroblasts. OLFML2B may influence various cancer and immune-related pathways, such as the PI3K-Akt signaling pathway, ECM–receptor interaction, focal adhesion, and leukocyte transendothelial migration. In addition, OLFML2B may increase drug resistance of binimetinib, cobimentinib, and trametinib.

**Conclusion:** Our outcomes reveal that OLFML2B may act as a prognostic marker and a potential target in immunotherapy for diverse tumors due to its oncogenesis function and immune infiltration.

## Introduction

Cancer is a complicated disease that is not confined to local tissue and metastasizes through the vascellum, lymph nodes, or transcoelomic seeding (Jin and Jin, 2020). Cancer has gradually become a serious public health problem worldwide and kills increasing number of people. There will be approximately 1,898,160 additions to cancer cases, the equivalent of 5,200 new cases every day in the United States in 2021 (Siegel et al., 2021). Although with the development of diagnosis and treatment technology, such as imaging technology and immunotherapy, there are still many cancer patients who cannot be cured because of lacking timely diagnosis and targeted drugs. Therefore, we need to find new methods to improve the cure rate.

The TME (tumor microenvironment) closely interacts with tumor cells to promote cancer progression, which consists of immunocytes, stromal cells, intercellular components, and metabolites located at the center, fringe, or within the surrounding of the tumor disorder (Jin and Jin, 2020). The TME is one of the vital factors that affect the efficacy of immunotherapy (Osipov et al., 2019). Hence, it is urgent to seek new biomarkers targeting the TME to ameliorate the effectiveness of immunotherapy.

Olfactomedin-like 2B (OLFML2B), as a member of the family of olfactomedin domain-containing proteins, is located on chromosomes 1q23.3 (Tomarev and Nakaya, 2009). It contains the unique Ser-/Thr-rich region preceding the olfactomedin domain, making it different from other family members and to form an independent subfamily of olfactomedin domain-containing proteins. OLFML2B is discovered in the ganglion cells and inner nuclear layers, the inner segment of the photoreceptor layer, and retinal pigmented epithelium (Furutani et al., 2005). Moreover, the prediction of destructive SNPs among the OLF genes illustrated that OLFML2B might cause the most harmful mutations (Li et al., 2019). However, only a few previous studies have focused on OLFML2B in cancer. OLFML2B, which is highly upregulated in GC, presents a moderate value of diagnosis and prognosis for GC (Liu et al., 2019) (Zhang et al., 2020) (Meng et al., 2020). In addition to gastric cancer, OLFML2B serves as a biomarker for diagnosis of HCC (Yang et al., 2020). Unfortunately, its function in other cancers, especially in the immune-related cancers, is still unclear and should be investigated in detail.

In the present research, we conducted a systematical analysis about the expression, predictive value, clinicopathology, genetic alteration, GSEA, TME, immune-associated gene, immune infiltration, MSI, TMB, and drug sensitivity in multiple cancer types. Our results have demonstrated that OLFML2B might have a potential value in tumor diagnosis and prognosis and serve as a marker for immunotherapy.

## Materials and Methods

### The Cancer Genome Atlas

The Cancer Genome Atlas (TCGA), a milestone program of cancer genomics, has molecularly characterized over 20,000 primary cancers and matched normal samples for 33 cancer types. We have downloaded OLFML2B expression data, clinical data, TMB data, and MSI data from the UCSC Xena online database (https://xenabrowser.net/).

### Oncomine

Oncomine is an extensively used database (https://www.oncomine.org) where we can evaluate the expression of the targeted genes. In the present study, we analyzed the expression of OLFML2B by setting filters such as gene symbol “OLFML2B,” the data type “mRNA,” cancer vs. normal, fold change “1.5,” *p*-value “0.05”, and gene rank “top 10%.”

### UALCAN

UALCAN is a comprehensive database (ualcan.path.uab.edu/home) where we can analyze the gene level and protein level. In this research, we analyzed the protein expression level of OLFML2B by the “CPTAC” module of UALCAN.

### Tumor Immune Estimation Resource

TIMER2.0 is a database (https://cistrome.shinyapps.io/timer/) of immune infiltration in multiple types of cancers. Our research has evaluated the expression of OLFML2B and the correlation between its expression and the extent of immune infiltration spanning 33 tumor types by the “Gene” module of TIMER2.0.

### Gene Expression Profiling Interactive Analysis

GEPIA is a database (http://gepia.cancer-pku.cn/) of the information about the targeted gene, such as expression, correlation, and survival. We have estimated the different expression of OLFML2B between tumor and normal tissues by the “Expression DIY” module through the TCGA database and GTEx database and OLFML2B similar genes by the “General” module of GEPIA.

### cBioPortal

cBioPortal is a database (https://www.cbioportal.org) of the multidimensional cancer genomics data. In the present research, we evaluated the copy number alteration (CNA), mutation status of OLFML2B, and prognosis across all TCGA tumors by the “Quick Search Beta!” module of cBioPortal.

### CellMiner

CellMiner is a drug-associated database (https://discover.nci.nih.gov/cellminer/home.do) based on the NCI-60 cell line set, which includes mRNA expression and drug sensitivity. In our study, we downloaded the related data by the “Download Data Sets” module of CellMiner to explore the relation between OLFML2B and drug sensitivity. We only selected the FDA-approved drugs and analyzed them by R software. *p* < 0.05 served as the standard of screening.

### Cell Culture

The MHCC-97H, HepG2, and Hep3B cell lines (acquired from the oncology laboratory of Tongji Hospital, Wuhan, China) were maintained in DMEM, and there was 10% FBS to complement DMEM. The LO2 cell line (gained from the oncology laboratory of Tongji Hospital) was cultured in RPMI-1640 medium with supplementary 10% FBS. The cells were maintained in a humidified 37°C incubator under 5% CO_2_ and 95% air. When cell confluence reached 80%, the cells were digested with 0.25% trypsin.

### cDNA Synthesis and Quantitative RT-PCR

Total RNA was extracted from the cells using TRIzol reagent (Takara, Shiga, Japan). The consistency and pureness were detected based on a NanoDrop 2000c UV spectrophotometer (Thermo Fisher Scientific), reading the protocol of the manufacturer for reference. The RevertAid First-Strand cDNA Synthesis Kit (Thermo Fisher Scientific, United States) and the Bio-Rad Laboratories S1000 Thermal Cycler (Hercules, CA) were applied to produce cDNA according to the total RNA. The amplification of cDNA samples was completed by making full use of Fast SYBR Green Master Mix (Thermo Fisher Scientific) on an ABI Prism 7900 Sequence Detector based on the protocol of the manufacturer (Applied Biosystems, Foster City, CA). According to SDS 2.1 software (Applied Biosystems), there was an automatic calculation for the baseline and threshold of the amplification curves. Melting curve analysis was carried out at the end of the program. The following primers were used: OLFML2B forward, 5′- AAG​CCT​CGG​CTG​CTA​GTT​C-3′ and reverse, 5′- GTT​GTC​CGC​CTC​GTT​TTG​C; GAPDH forward, 5′- GGA​GCG​AGA​TCC​CTC​CAA​AAT-3′ and reverse, 5′- GGC​TGT​TGT​CAT​ACT​TCT​CAT​GG-3’. GAPDH was regarded as an internal reference. The relative gene expression levels were computed based on the comparative cycle threshold approach. There were three duplicated PCR amplifications for a single sample.

### Tissue Specimens and Immunohistochemistry

Forty-four liver cancer primary tissue samples and adjacent matched normal tissue samples were used to explore the protein expression level of OLFML2B. Tissue sections were incubated with primary antibody (OLFML2B, YT3706, 1:100, Immunoway). The protocol was presented by Huang et al., (2017).

### Statistical Analysis

The association of OLFML2B expression with TMB, MSI, and immune-related genes was analyzed by the Spearman’s test or Pearson’s test, and the analysis of the difference in multiple type cancers was analyzed by the Wilcoxon test. Survival curves were compared using log-rank tests. *p* < 0.05 was estimated to be statistically significant. The gene expression data were subjected to log2 transformative normalization. All the statistical analyses were calculated on R software.

## Results

### The Analysis of OLFML2B Expression

**FIGURE 1:**
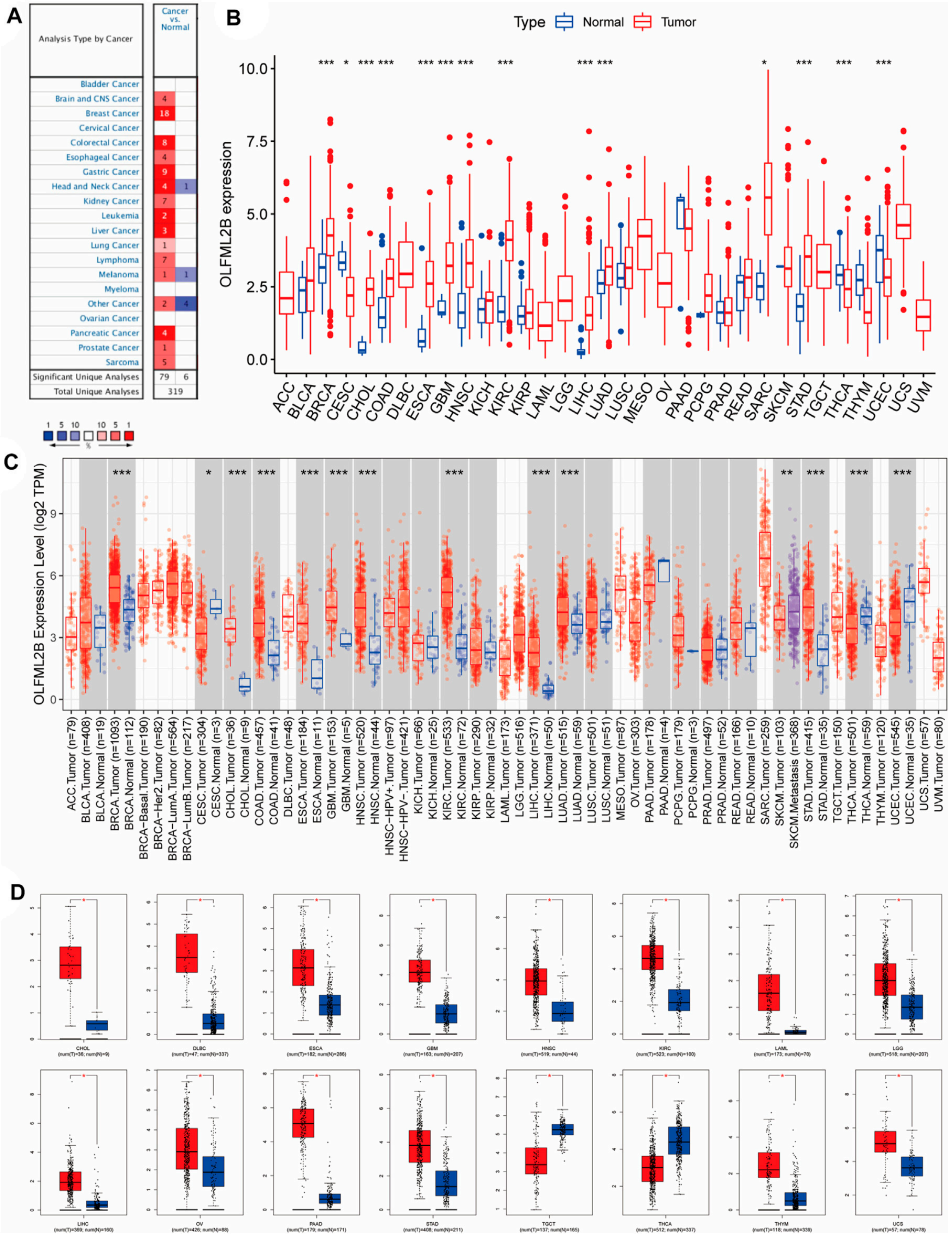
Pan-cancer OLFML2B expression level. **(A)** Expression level of OLFML2B in the Oncomine database. **(B)** Expression level of OLFML2B in the TCGA database. **(C)** Expression level of OLFML2B in the TIMER2.0 database. **(D)** Expression level of OLFML2B in the GEPIA database.

We investigated the expression level of OLFML2B from 11,057 samples of tumor and normal tissues (10,327 tumor and 730 normal samples) based on the TCGA database. Our outcomes suggested that OLFML2B was significantly upregulated or downregulated in several cancers. OLFML2B was overexpressed in BRCA, CHOL, COAD, ESCA, GBM, HNSC, KIRC, LIHC, LUAD, SARC, and STAD, while it was underexpressed in CESC, THCA, and UCES when compared between the tumor and normal samples (Figure 1B). The abbreviations and the flow sheet diagram are explained in Supplementary Figure S1. Moreover, OLFML2B was highly expressed in metastatic SKCM tissue when compared to the primary tumor tissue based on TIMER2.0 (Figure 1C). In addition, we found that OLFML2B was upregulated in most cancers except bladder cancer, cervical cancer, myeloma, and ovarian cancer, according to the Oncomine database. Meanwhile, the downregulation of OLFML2B was observed in head and neck cancer and melanoma (Figure 1A).

Due to normal tissue shortages in the TCGA database, we then applied GEPIA to explore the expression of OLFML2B, which matched with the GTEx database to increase the number of normal tissues. The results illustrated that OLFML2B was upregulated in 14 cancers and downregulated in two cancers (Figure 1D).

**FIGURE 2:**
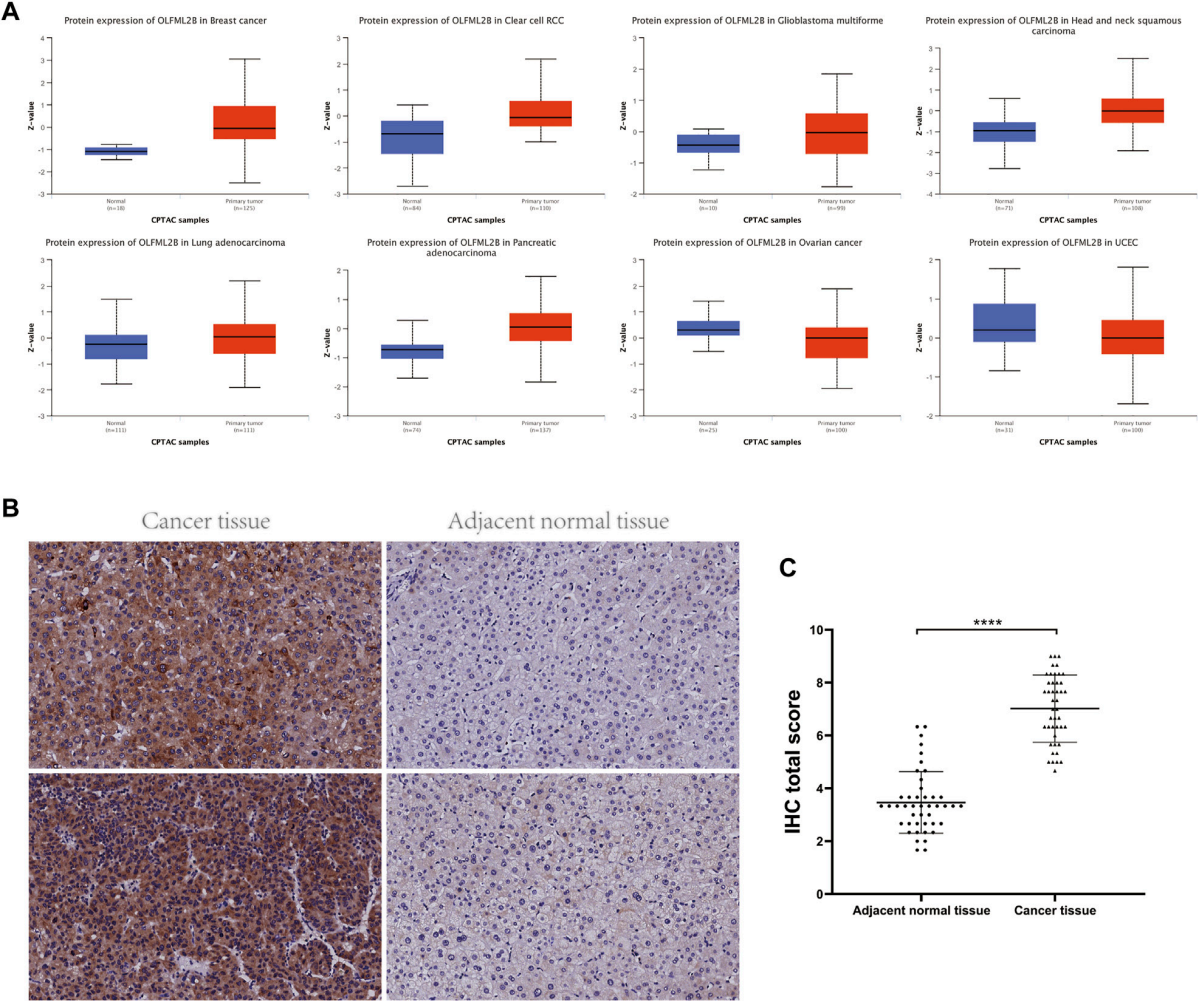
Protein expression level and prognostic value of OLFML2B. **(A)** Protein expression level of OLFML2B in the UALCAN database. **(B)** Higher OLFML2B expression and lower OLFML2B expression in the human liver cancer tissues and adjacent normal tissues. **(C)** H-score of OLFML2B IHC staining of cancer tissues and corresponding adjacent normal tissues.

Furthermore, we analyzed the protein expression of OLFML2B based on the CPTAC database applying UALCAN tools. OLFML2B protein was increased in BRCA, HNSC, KIRC, LGG, LUAD, and PAAD. Nevertheless, OLFML2B was decreased in OV and UCEC (Figure 2A). There existed some paradoxes in the expression of OLFML2B through different databases due to diverse data sources and analysis methods.

According to the abovementioned pan-cancer research, we further concentrated on the expression level of OLFML2B in the LIHC cell lines (Supplementary Figure S2). The outcomes from qPCR indicated that the expression of OLFML2B was upregulated in the LIHC cell lines (MHCC-97H, HepG2, and Hep3B) when compared with the normal human hepatocytes (LO2). In addition, we verified the expression level of OLFML2B in liver cancer tissues by immunohistochemistry, and the results were consistent with those of the database (Figures 2B,C).

### The Analysis of the Prognostic Value of OLFML2B

**FIGURE 3:**
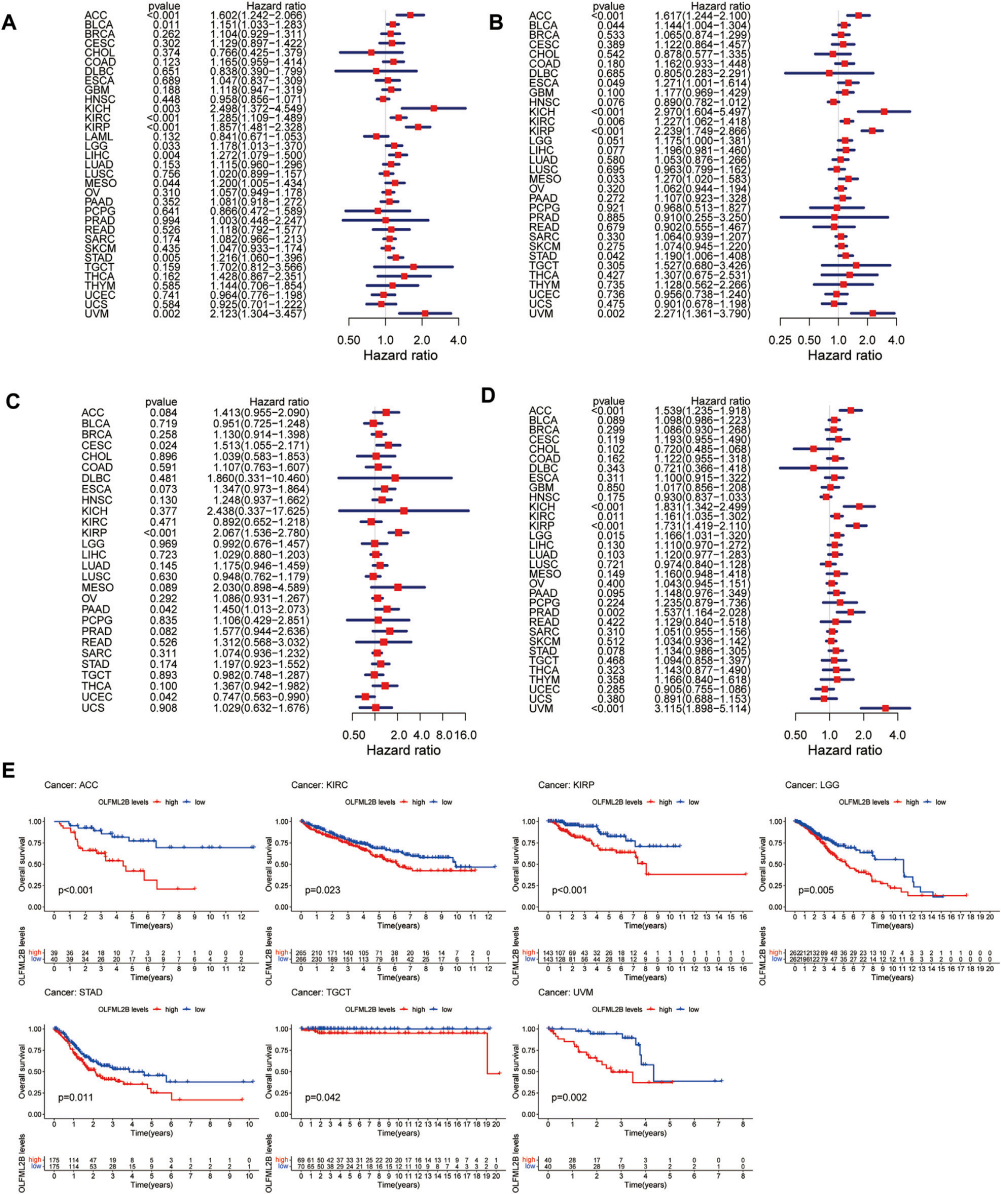
Prognostic value of OLFML2B. **(A)** Forest plot of OS correlation in pan-cancer. **(B)** Forest plot of DSS correlation in pan-cancer. **(C)** Forest plot of DFI correlation in pan-cancer. **(D)** Forest plot of PFI correlation in pan-cancer. **(E)** KM analysis of the association of OLFML2B and OS.

To evaluate the association between the expression level of OLFML2B and prognostic value, we performed several metrics based on R software, consisting of OS, DSS, DFI, and PFI. Cox analysis revealed that OLFML2B expression was apparently correlated with OS in ACC (HR = 1.602), BLCA (HR = 1.151), KICH (HR = 2.498), KIRC (HR = 1.285), KIRP (HR = 1.857), LGG (HR = 1.178), LIHC (HR = 1.272), MESO (HR = 1.200), STAD (HR = 1.216), and UVM (HR = 2.123) (Figure 3A). In addition, KM analysis suggested that upregulation of OLFML2B was associated with poorer survival in ACC, KIRC, KIRP, LGG, STAD, TGCT, and UVM (Figure 3E).

When it comes to DSS, Cox analysis indicated that OLFML2B was remarkably correlated with DSS in ACC (HR = 1.617), BLCA (HR = 1.144), ESCA (HR = 1.271), KICH (HR = 2.970), KIRC (HR = 1.227), KIRP (HR = 2.239), MESO (HR = 1.270), STAD (HR = 1.190), and UVM (HR = 0.002) (Figure 3B). Furthermore, KM analysis showed that overexpression of OLFML2B was associated with worse survival in ACC, ESCA, KIRC, KIRP, LGG, LIHC, and UVM (Supplementary Figure S3A).

As for DFI, Cox analysis illustrated that OLFML2B expression was notably correlated with DFI in ACC (HR = 1.617), BLCA (HR = 1.144), ESCA (HR = 1.271), KICH (HR = 2.970), KIRC (HR = 1.227), KIRP (HR = 2.239), MESO (HR = 1.270), STAD (HR = 1.190), and UVM (HR = 2.271) (Figure 3C). Furthermore, KM analysis demonstrated that upregulation of OLFML2B was associated with decreased DFI in CESC and KIRP (Supplementary Figure S3B).

With reference to PFI, Cox analysis revealed that the expression of OLFML2B is strikingly correlated with PFI in ACC (HR = 1.539), KICH (HR = 1.831), KIRC (HR = 1.161), KIRP (HR = 1.731), LGG (HR = 1.166), PRAD (HR = 1.537), and UVM (HR = 3.115) (Figure 3D). Furthermore, KM analysis showed that OLFML2B upregulation was associated with diminished PFI in ACC, KIRC, KIRP, LGG, PRAD, and UVM (Supplementary Figure S3C).

### Correlation Between OLFML2B Expression and Clinicopathology in Multiple Cancer Types

**FIGURE 4:**
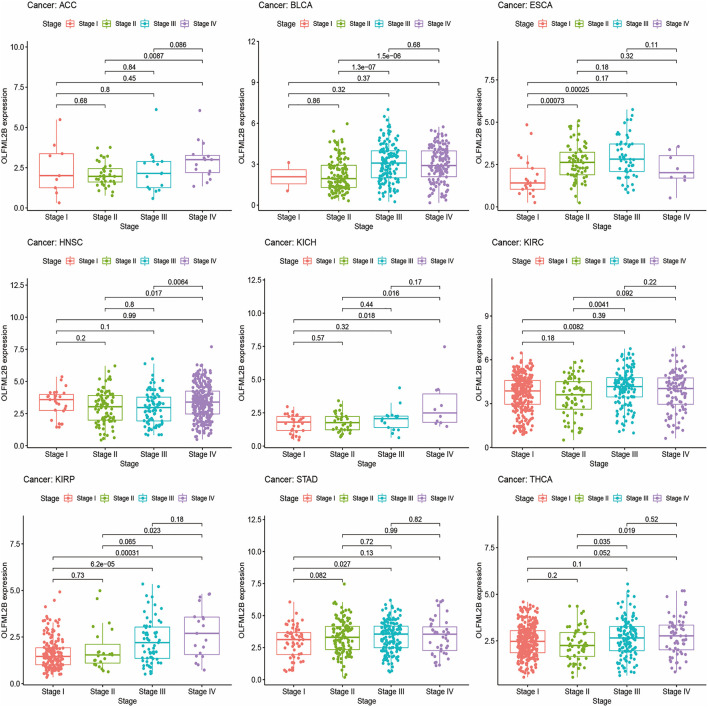
Correlation between the expression level of OLFML2B and cancer stage.

We explored the correlation between OLFML2B expression and cancer stage and then detected that the expression level of OLFML2B was associated with tumor stage in ACC, BLCA, ESCA, HNSC, KICH, KIRC, KIRP, STAD, and THCA (Figure 4). There existed obvious difference in ACC, BLCA, KICH, KIRC, KIRP, and STAD between early stages (stage Ⅰ and stage Ⅱ) and advanced stages (stage Ⅲ and stage Ⅳ), and the expression level of OLFML2B is higher in advanced stages than in early stages. However, OLFML2B expression was gradually increased from stage Ⅰ to stage Ⅲ and decreased in stage Ⅳ in ESCA. As in THCA, OLFML2B was upregulated from stage Ⅱ to stage Ⅳ.

### Alteration Frequency Level of OLFML2B

**FIGURE 5:**
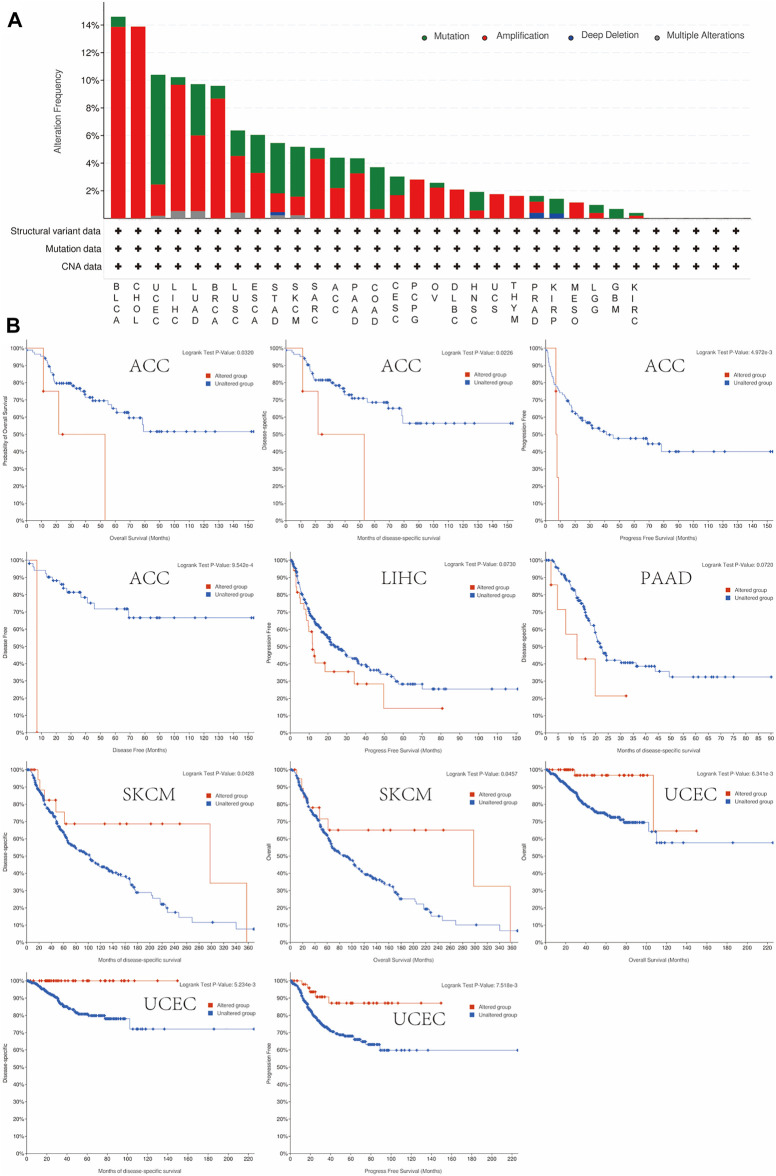
Mutation feature of OLFML2B in pan-cancer. **(A)** Mutation status of OLFML2B in pan-cancer. **(B)** Correlation between the prognosis and alteration in ACC, LIHC, SKCM, and UCEC.

DNA copy number alteration is an essential factor affecting the expression of protein-coding and noncoding genes and influencing the activity of various signaling pathways (Liang et al., 2016). We evaluated the copy number alteration (CNA) and mutation status of OLFML2B, and the results revealed that the top five cancer types with total mutations were BLCA, CHOL, UCEC, LIHC, LUAD, and BRCA (Figure 5A). The most common mutation status was amplification, especially in BLCA, CHOL, LIHC, and BRCA (Figure 5A). Then, we investigated the correlation between prognosis and alteration in tumors. We found that the altered group was correlated to poor prognosis in ACC, LIHC, and PAAD than the nonaltered group (Figure 5B). However, the altered and non-altered groups in LIHC (*p* = 0.072) and PAAD (*p* = 0.073) for prognosis were not statistically significant, possibly because of the small sample size. Then, we discovered that the altered group was correlated to a better prognosis in SKCM and UCEC than the non-altered group (Figure 5B).

### OLFML2B-Associated Gene Enrichment Analysis and Protein–Protein Interaction Network

**FIGURE 6:**
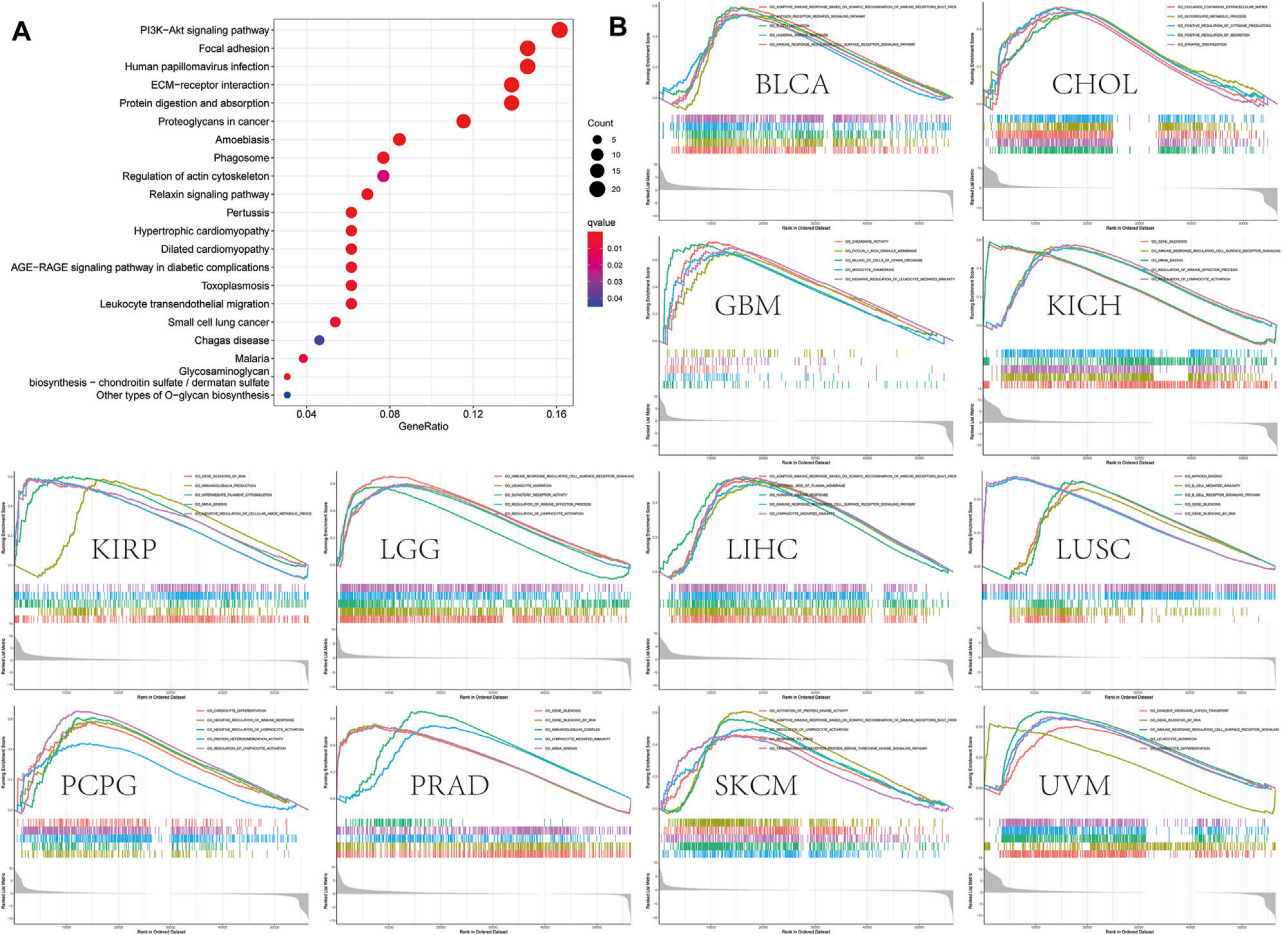
OLFML2B-associated gene enrichment analysis. **(A)**KEGG pathway analysis of OLFML2B-correlated genes. **(B)**GO analysis of OLFML2B.

We selected 400 genes that were similar to OLFML2B from GEPIA based on the TCGA database to research the function and pathway of OLFML2B. The analysis of the KEGG pathway revealed that OLFML2B could modulate several tumorigeneses and immune pathways, such as “PI3K-Akt signaling pathway,” “focal adhesion,” “ECM–receptor interaction,” and “leukocyte transendothelial migration” (Figure 6A). In addition, we performed the analysis of GO functional annotations to investigate the function of OLFML2B based on the “limma” algorithm, “clusterProfiler” algorithm, and “enrichplot” algorithm. We discerned that OLFML2B regulated “the immune response regulating cell surface receptor signaling pathway” in BLCA, KICH, LGG, LIHC, THCA, and UVM; “lymphocyte activation” in LGG, KICH, PCPG, and SKCM; and “gene silencing” in KIRP, LUSC, PRAD, THYM, and USC (Figure 6B). In addition, OLFML2B may regulate several cancer-associated functions, and the detailed information is displayed in Supplementary Figures S4, S5. So, we conjectured that OLFML2B played an essential role in cancer progression and tumor immune microenvironment.

**FIGURE 7:**
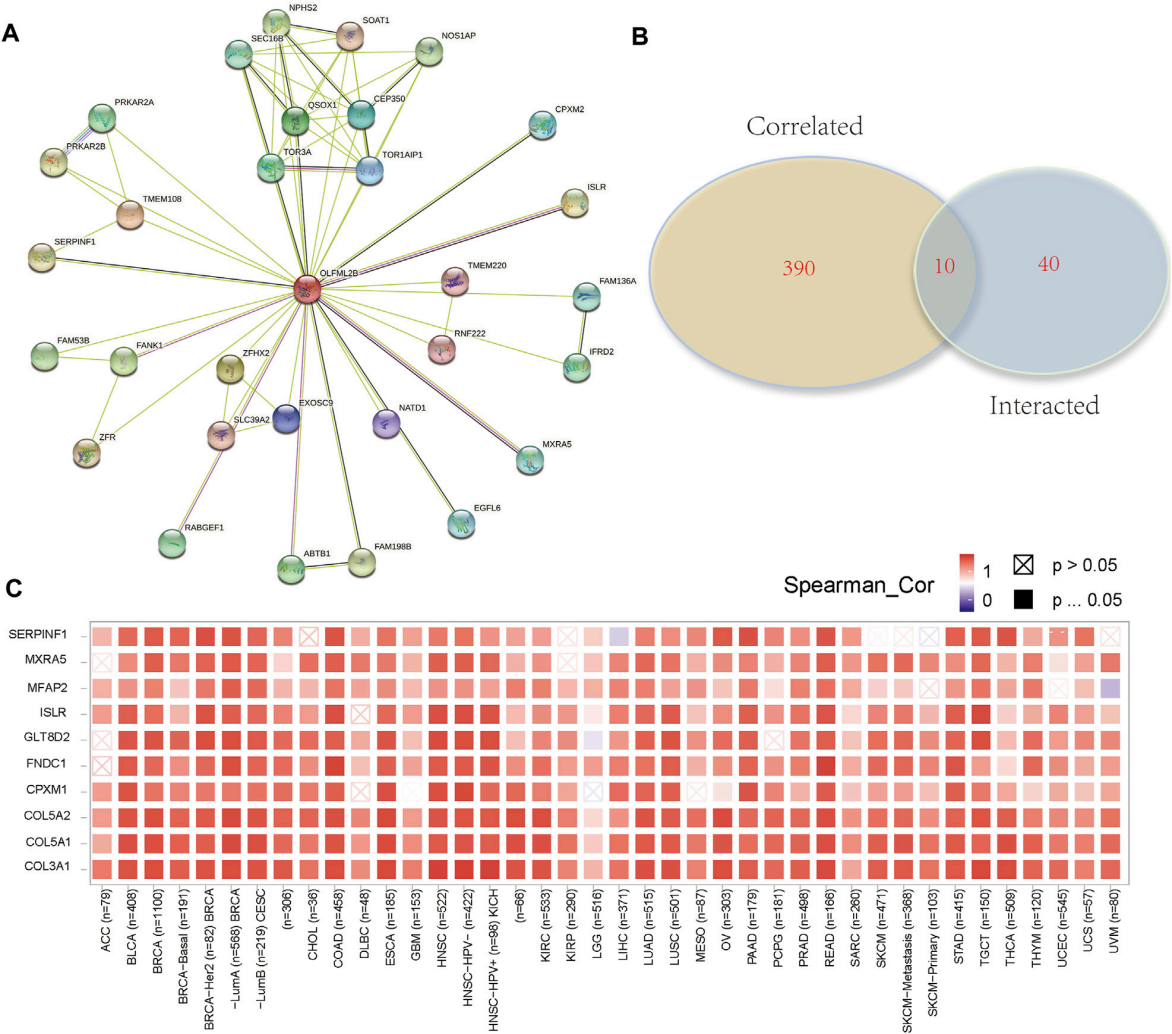
OLFML2B-associated gene enrichment analysis. **(A)**PPI network of OLFML2B. **(B)** Venn diagram analysis. **(C)** Heatmap of intersected genes.

To evaluate the molecular mechanisms of OLFML2B in tumor immunity and oncogenesis, we carried out an analysis of the PPI network of OLFML2B (Figure 7A). We gained 50 OLFML2B-binding proteins based on STRING. Then, we conducted a Venn diagram analysis of the two groups from GEPIA and STRING to show the same genes (Figure 7B), and a heatmap of these genes was constructed to present the positive correlation (Figure 7C). Most of these genes have been proven to promote tumorigenesis.

### Association Between OLFML2B Expression and TME in Multiple Tumors

**FIGURE 8:**
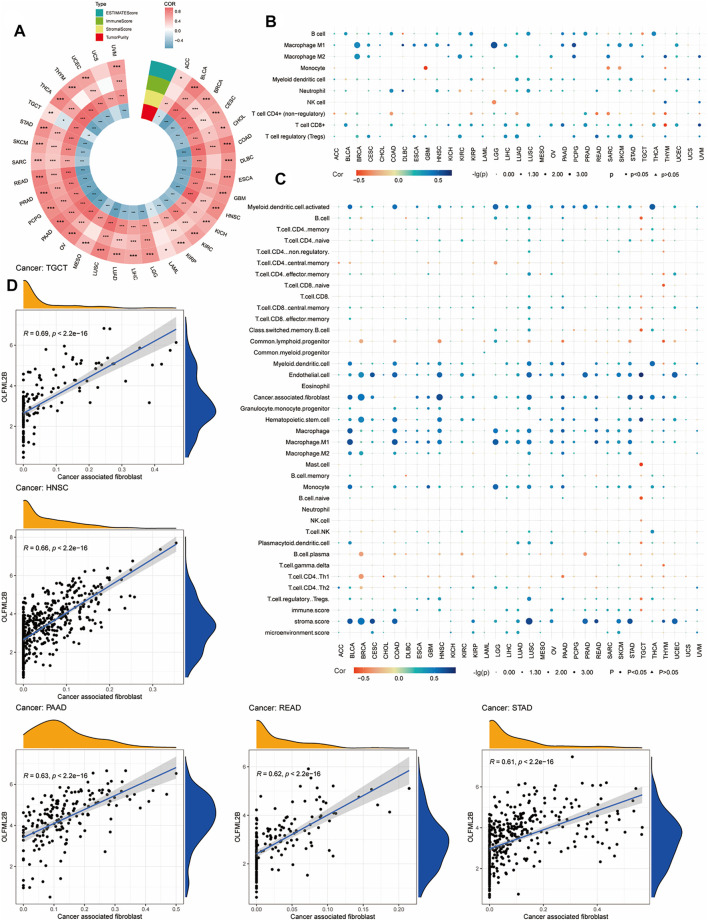
Association between OLFML2B and TME and immune infiltration. **(A)** Association between OLFML2B expression and TME in pan-cancer. **(B)** Correlation between OLFML2B expression and immune infiltration based on QUANTISEQ algorithms. **(C)** Correlation between OLFML2B expression and immune infiltration based on XCELL algorithms. **(D)** Five tumors with the highest correlation between OLFML2B and CAFs.

Numerous pieces of evidence have suggested that the TME plays a crucial role in the development and progression of tumors (Osipov et al., 2019). Consequently, it was necessary to investigate the association between the expression level of OLFML2B and TME based on the ESTIMATE algorithm to calculate the stromal scores, immune scores, and tumor purity for pan-cancer. The outcomes indicated that the expression level of OLFML2B is positively correlated with the stromal scores in all types of cancers and immune scores in 24 cancers. Furthermore, OLFML2B expression was negatively associated with immune scores in TGCT and tumor purity in all types of cancers except UCS (Figure 8A).

### The Analysis of Immune Infiltration of OLFML2B

Due to the significant association between OLFML2B and TME in almost all types of cancer, we researched the correlation between OLFML2B expression and immune infiltration based on the “immunedeconv” package in R software (XCELL and QUANTISEQ algorithms). The findings have indicated that there is a positive association between OLFML2B expression and CAFs (cancer-associated fibroblast), M2 (macrophage2), and Tregs (T cell regulatory) (Figures 8B,C). Meanwhile, we discerned that the upregulation of OLFML2B was positively correlated to CAFs in 23 cancers (Figure 8C). In the association between the expression of OLFML2B and Tregs, M2 was different in the same types of cancers based on various algorithms, possibly owing to the heterogeneous principles. No matter which kind of algorithms we chose, there still existed diverse types of cancers, where we could find a significant relation between the expression of OLFML2B and immune infiltration.

### Correlation Between OLFML2B Expression and Immune-Associated Genes in Different Tumors

**FIGURE 9:**
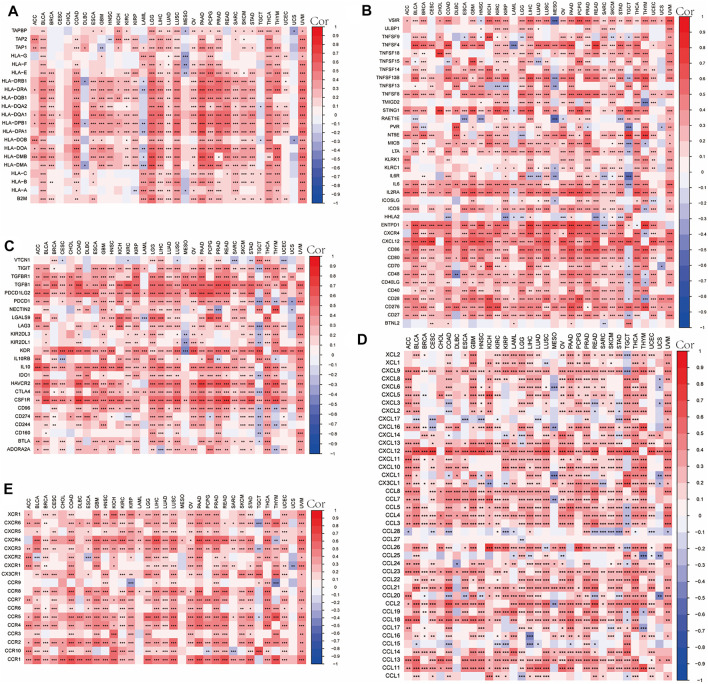
Correlation between OLFML2B expression and immune-associated genes in pan-cancer. **(A)** MHC genes heatmap. **(B)** Immune active genes heatmap. **(C)** Immunosuppressive genes heatmap. **(D)** Chemokine genes heatmap. **(E)** Chemokines receptor genes heatmap.

We analyzed gene coexpression to research the correlation between OLFML2B expression and immune-associated genes in 33 types of cancer, such as chemokines genes, chemokine receptor genes, immune activation genes, immunosuppressive genes, and MHC genes. The results about chemokine genes suggested that almost all of these are coexpressed with OLFML2B except CCL27(Figure 9D). The outcomes about chemokine receptor genes indicated that OLFML2B expression was related to these in all types of cancers, except UCS (Figure 9E). Moreover, there exists a coexpression between OLFML2B and immune activation genes, especially in TNFSF4, TNFSF8, STING1, IL6, IL2RA, CXCR4, CXCL12, CD276, and CD27 (Figure 9B). In addition, the immunosuppressive genes were equally coexpressed with OLFML2B, such as TIGIT, TGFB1, IL10, CTLA4, CSF1R, CD274, and BTLA (Figure 9C). Furthermore, the coexpression analysis between OLFML2B and MHC genes illustrated that the coexpression relationship existed in diverse cancers, except CESC, CHOL, and UCS (Figure 9A).

### Correlation Between OLFML2B Expression and TMB or MSI in Several Tumors

**FIGURE 10:**
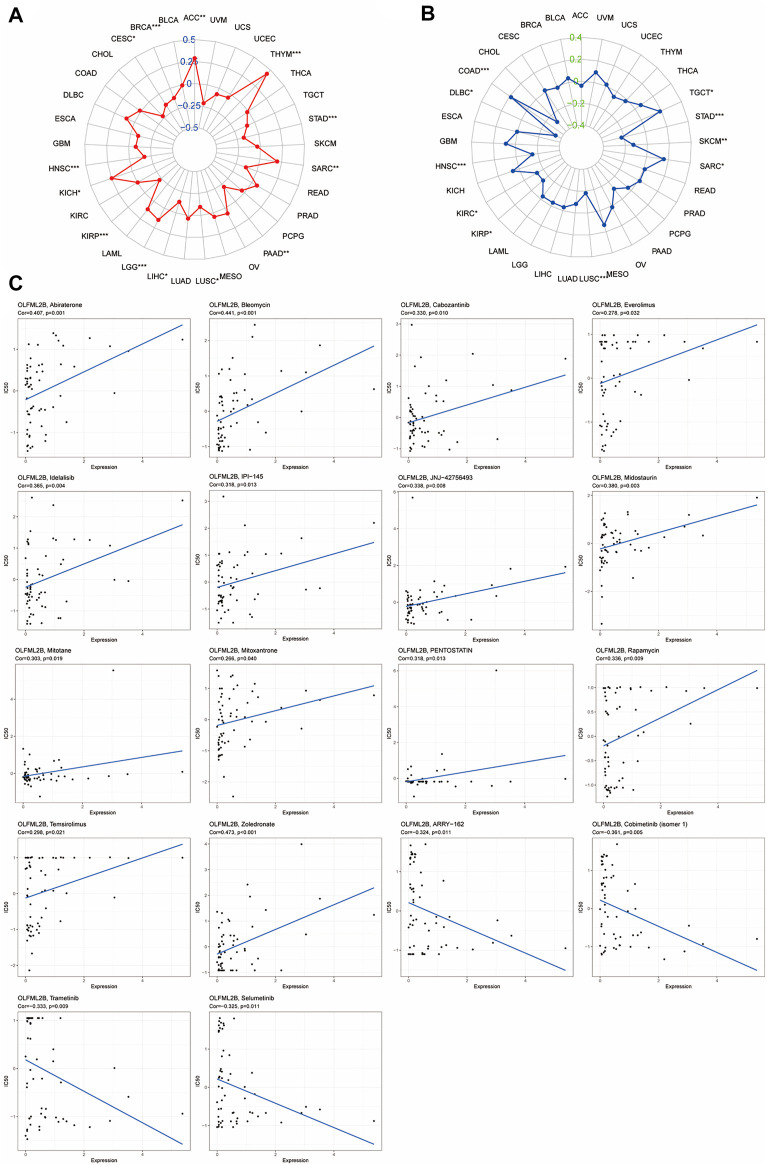
Correlation between OLFML2B expression and TMB, MSI, and drug sensitivity. **(A)** Radar map of correlation between OLFML2B and TMB. **(B)** Radar map of correlation between OLFML2B and MSI. **(C)** Correlation between OLFML2B expression and drug sensitivity.

According to previous research, MSI and TMB may be a predictor to ICIs (immune checkpoint inhibitors) (Sahin et al., 2019; Addeo et al., 2021). Therefore, we investigated the correlation between OLFML2B and TMB or MSI in different cancers based on R software. The outcomes have indicated that a high expression level of OLFML2B was positively associated with TMB in ACC, KICH, LGG, SARC, and THYM. However, it was negatively correlated with TMB in BRCA, CESC, HNSC, KIRP, LIHC, LUSC, PAAD, and STAD (Figure 10A). Moreover, the results have also revealed that upregulation of OLFML2B was positively associated with MSI in COAD, SARC, and TGCT. On the contrary, it was negatively correlated with MSI in DLBC, HNSC, KIRC, KIRP, LUSC, SKCM, and STAD (Figure 10B).

### Correlation Between Drug Sensitivity and OLFML2B Expression

We estimated the association between drug sensitivity and OLFML2B expression by the CellMiner database. Only the FDA-approved drugs were included in the analysis. The overexpression of OLFML2B could improve the drug sensitivity of abiraterone, bleomycin, cabozantinib, everolimus, idelalisib, IPI-145 (duvelisib), JNJ-42756493 (erdafitinib), midostaurin, mitotane, mitoxantrone, pentostatin, rapamycin, temsirolimus, and zoledronate (Figure 8C). The upregulation of OLFML2B could impair the drug sensitivity of ARRY-162 (Binimetinib), cobimentinib (isomer1), selumetinib, and trametinib (Figure 10C).

## Discussion

OLFML2B, one of the olfactomedin domain-containing protein families, exerts a significant role specifically linking to chondroitin sulfate-E or heparin in the extracellular matrix (Furutani et al., 2005). According to previous studies, OLFML2B is associated with perineural invasion in HNSCC (head and neck squamous cell carcinoma) (Zhang et al., 2019). In addition, OLFML2B has been proven to be upregulated, presented a diagnostic and prognostic value (Liu et al., 2019), and correlated with the TME in GC and HCC (Ren et al., 2020) (Liu et al., 2020). Furthermore, OLFML2B is bound up with tumorigenesis and prognosis in bladder cancer (Zhao et al., 2020). OLFML2B can serve as a potential prognostic biomarker for osteosarcoma (Yao et al., 2021). On account of former research, we suspect that OLFML2B may influence the development of tumor to change the prognosis. However, there is no systematic pan-cancer analysis of OLFML2B, especially in tumor immune response, and we do not understand the role of OLFML2B in tumor immune response.

As a result, we perform this research as the first pan-cancer analysis of OLFML2B to investigate its function, such as in prognosis, clinicopathology, pathways, TME, immune infiltration, immune-associated genes, MSI, TMB, and drug sensitivity. OLFML2B overexpresses in multiple cancers, which illustrates that OLFML2B may promote the development of tumors. The results of OS, DSS, DFI, and PFI demonstrate that upregulation of OLFML2B probably indicates poorer prognosis in diverse tumors, especially in AAC, ESCA, KIRC, KIRP, LGG, LIHC, STAD, and UVM. High expression of OLFML2B indicates advanced stages in ACC, BLCA, KICH, KIRC, KIRP, and STAD. Our results are consistent with those of previous research and could be a supplement.

Therefore, we conduct the gene enrichment analysis of OLFML2B to explore the function and signaling pathways. KEGG pathway analysis supports these viewpoints as well. OLFML2B may activate a number of cancer-associated pathways, such as PI3K-Akt signaling pathway, focal adhesion, ECM–receptor interaction, proteoglycans in cancer, and leukocyte transendothelial migration, which indicates that OLFML2B may promote tumorigenesis, migration, and regulate tumor immune response. GO functional annotations demonstrate that OLFML2B regulates the immune system, such as lymphocyte activation, immune response regulating cell surface receptor signaling pathway, and B cell activation. Based on these outcomes, we conjecture that OLFML2B promotes tumor progression by influencing tumor immune response.

The tumor microenvironment conspicuously exerts an influence on treatment response and clinical outcomes (Wu and Dai, 2017). The results of the TME analysis certificate that overexpression of OLFML2B is positively related to stromal scores and immune scores but negatively associated with tumor purity in almost all cancers. Meanwhile, OLFML2B is related to immune infiltration, especially in Tregs and M2 in ESCA, KIRC, LIHC, STAD, and LGG, which also indicates that OLFML2B could change tumor immune response and may be a biomarker for immunotherapy. The results of immune infiltration agree with those of the TME. The upregulation of OLFML2B increases the stromal scores by recruiting cancer-associated fibroblasts and other stromal cells, which have been proven to promote tumorigenesis, tumor angiogenesis, neoplasm metastasis, and drug resistance, especially in ESCA, KIRC, LIHC, STAD, and LGG. Previous research has shown that TGF-β and IL-6 are related to the appearance of CAF phenotypes with the strengthened capacity for synthetizing and secretion (Chen and Song, 2019). Interestingly, there exists a positive coexpression between OLFML2B and IL-6 and TGF-β, which suggests that OLFML2B may promote the recruitment of CAFs through TGF-β and IL-6. The overexpression of OLFML2B increases the immune scores by upregulation of M2, which has been shown as a tumor promoter to improve the ability of tumor migration and immune escape. Experimental research has illustrated that CCL2 promotes M2 recruitment and turns monocytes into M2-polarized macrophages (Zhou et al., 2020). Knockdown of CCL2 in tumor cell lines remarkably impairs tumorigenesis with downregulation of TAM infiltration. CSF1, CCL5, and CXCL12 also present the function of recruitment of M2 besides CCL2 (Chanmee et al., 2014). The coexpression analysis of OLFML2B reveals a positive coexpression between OLFML2B and CCL2, CCL5, CXCL12, and CSF1, which support the initial point. Furthermore, we discovered that OLFML2B is associated with the infiltration of Tregs in several cancers, especially in ESCA, KIRC, KIRP, LIHC, STAD, and UVM. Tregs possess powerful immune suppressive ability and weaken antitumor immune efficiency in tumor-bearing hosts. A previous study has shown that CCR4 is a vital chemokine receptor responsible for Tregs migration to the TME in response to chemokines (Ohue and Nishikawa, 2019). CCR4 is bound by CCL22, which is positively coexpressed with OLFML2B in 22 cancers. According to this, we speculate that OLFML2B may recruit Tregs through CCL22.

CAFs and TAMs could promote drug resistance to cause poor prognosis in many cancers (Nurmik et al., 2020) (Nowak and Klink, 2020). Consequently, we indagate the effect of OLFML2B on drug sensitization. The upregulation of OLFML2B could impair the drug sensitivity of binimetinib, cobimentinib, and trametinib. All of these drugs are inhibitors of mitogen-activated protein kinase, which are used to treat melanoma. Therefore, there may be an improvement in the therapeutic effect of melanoma by targeting OLFML2B. Moreover, the expression of OLFML2B is bound up with TMB in 13 tumors and MSI in 10 tumors, which indicates that OLFML2B possibly influences the effectiveness of treatments for ICI. Thus, the expression level of OLFML2B may be used as an indicator for antitumor therapy.

In summary, the results of systematical pan-cancer analysis of OLFML2B may corroborate the association between its expression and cancers from several aspects. Consequently, OLFML2B may serve as a predictor of prognosis based on immunosuppression, and targeting it may be a novel therapeutic approach.

However, the research has some limitations. On one hand, there are few cases of some types of cancers, contributing to the imprecise analysis outcomes, and there may be a batch effect. On the other hand, the lack of experimental research indicates that this study may be a preliminary work. Furthermore, we need to perform exploratory research to test and verify.

## Conclusion

OLFML2B is extensively upregulated in numerous cancers. In addition, its overexpression is associated with poor prognosis, advanced stage of tumor, and transformation of TME and immune infiltration. Thus, OLFML2B may be used as a new target for cancer treatment.

## Data Availability

Publicly available datasets were analyzed in this study. The datasets can be found here: CellMiner database (https://discover.nci.nih.gov/cellminer/home.do) and UCSC Xena database (https://xenabrowser.net/).

## Supplementary Material

Supplementary Figure S1
**(A)** Explanation of abbreviations. **(B)** Flow sheet diagram.

Supplementary Figure S2 Relative expression of OLFML2B in LIHC cell lines.

Supplementary Figure S3 Prognostic value of OLFML2B. **(A)** KM analysis of the association of OLFML2B and DSS. **(B)** KM analysis of the association of OLFML2B and DFI. **(C)** KM analysis of the association of OLFML2B and PFI.

Supplementary Figure S4 Results of GO analysis.

Supplementary Figure S5 Results of GO analysis.
