# Supplementary figures and images for: Pan-Cancer Analysis of OLFML2B Expression and Its Association With Prognosis and Immune Infiltration
Source: Front Genet. 2022 Jul 6;13:882794. doi: 10.3389/fgene.2022.882794 (PMC9298975; doi:10.3389/fgene.2022.882794)

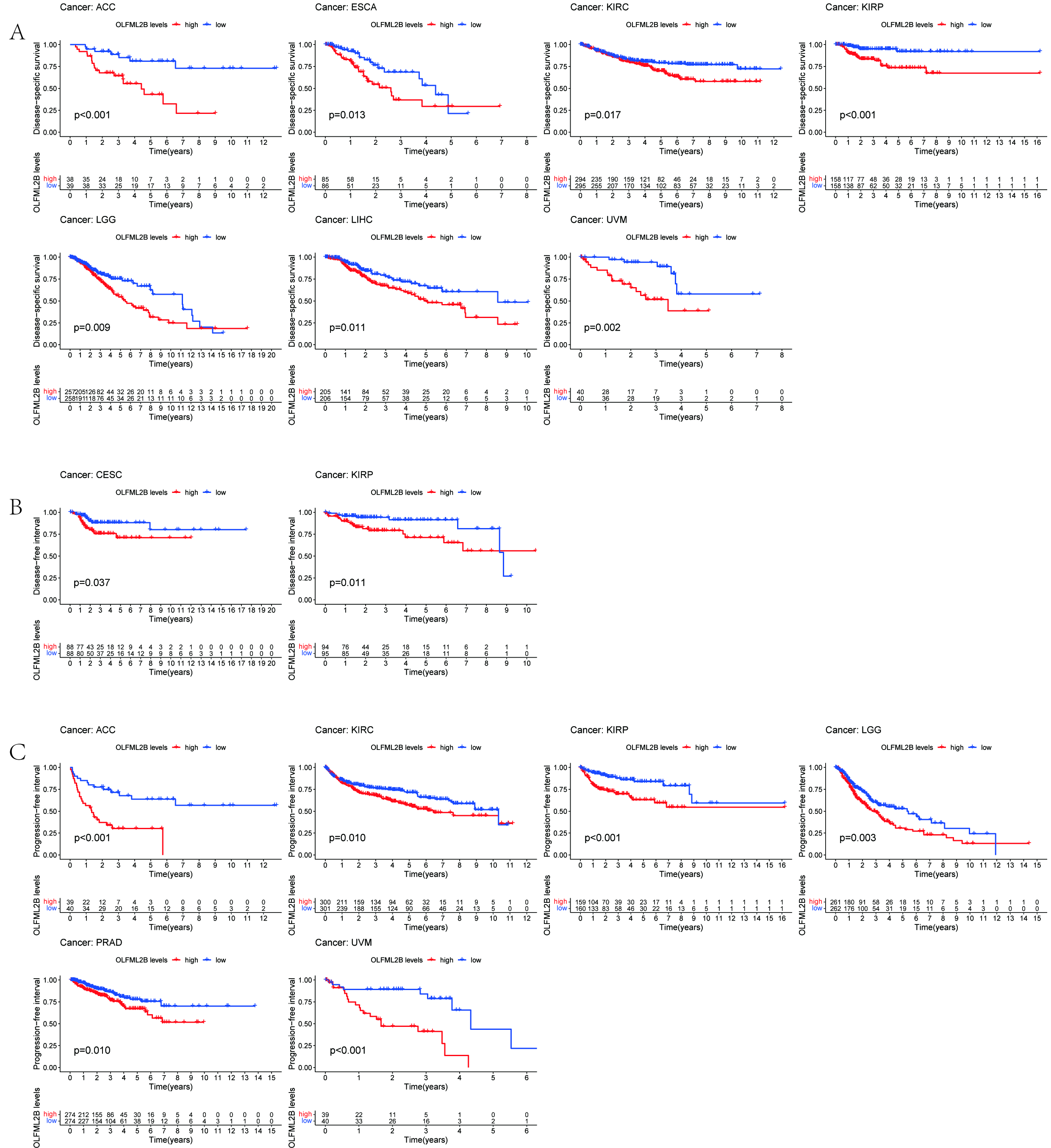

Supplement: Supplementary file 1 [file Image3.TIF]

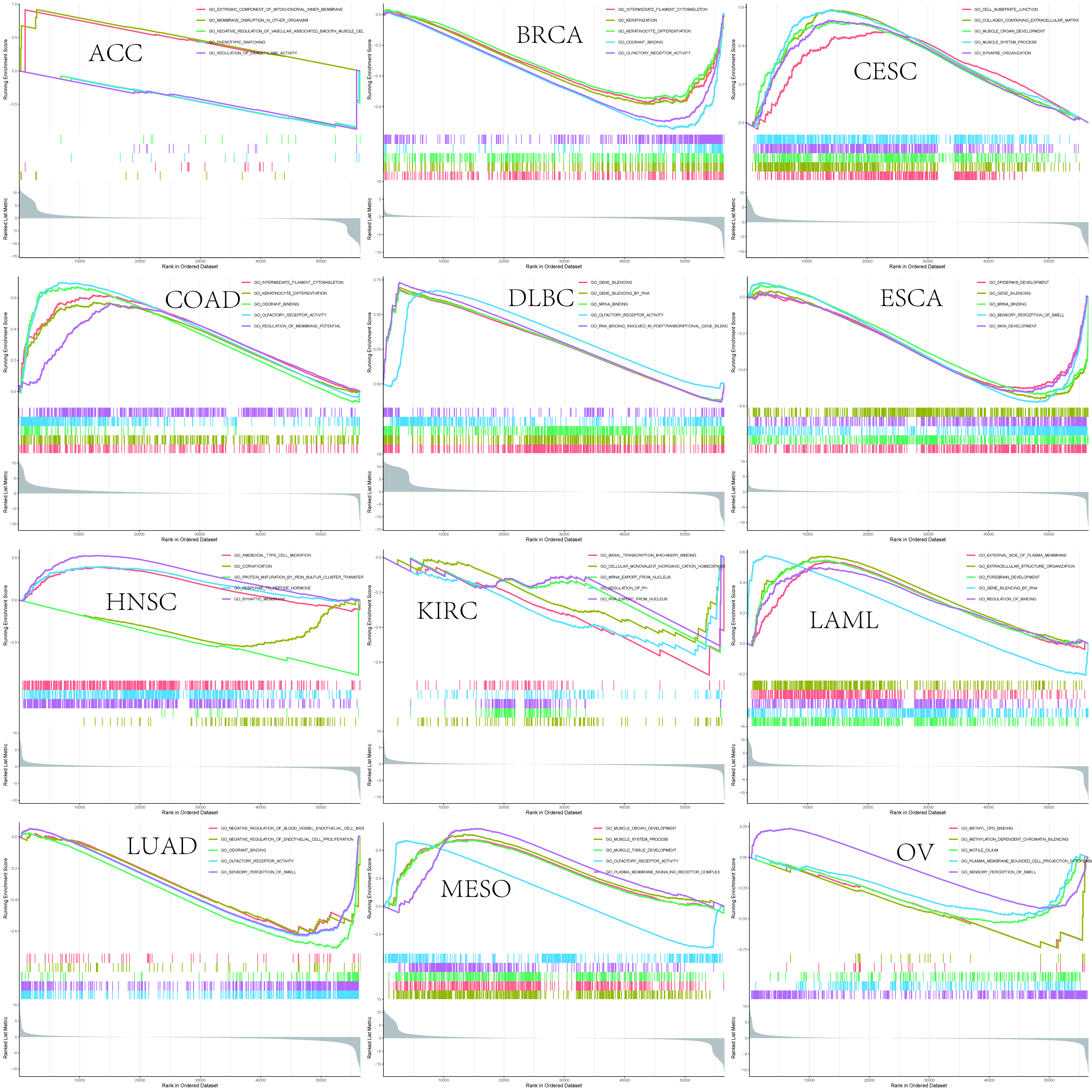

Supplement: Supplementary file 2 [file Image4.TIF]

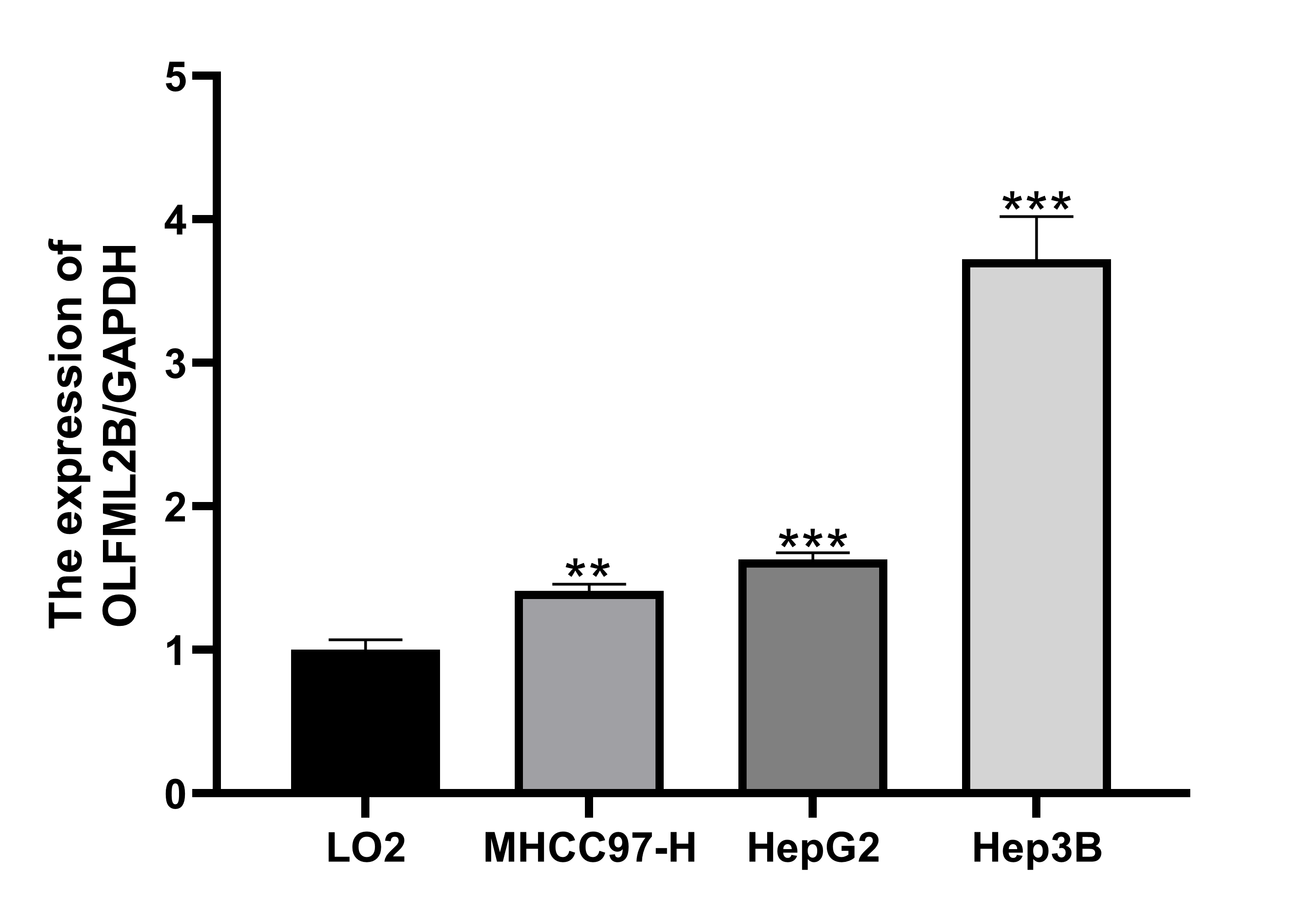

Supplement: Supplementary file 3 [file Image2.TIF]

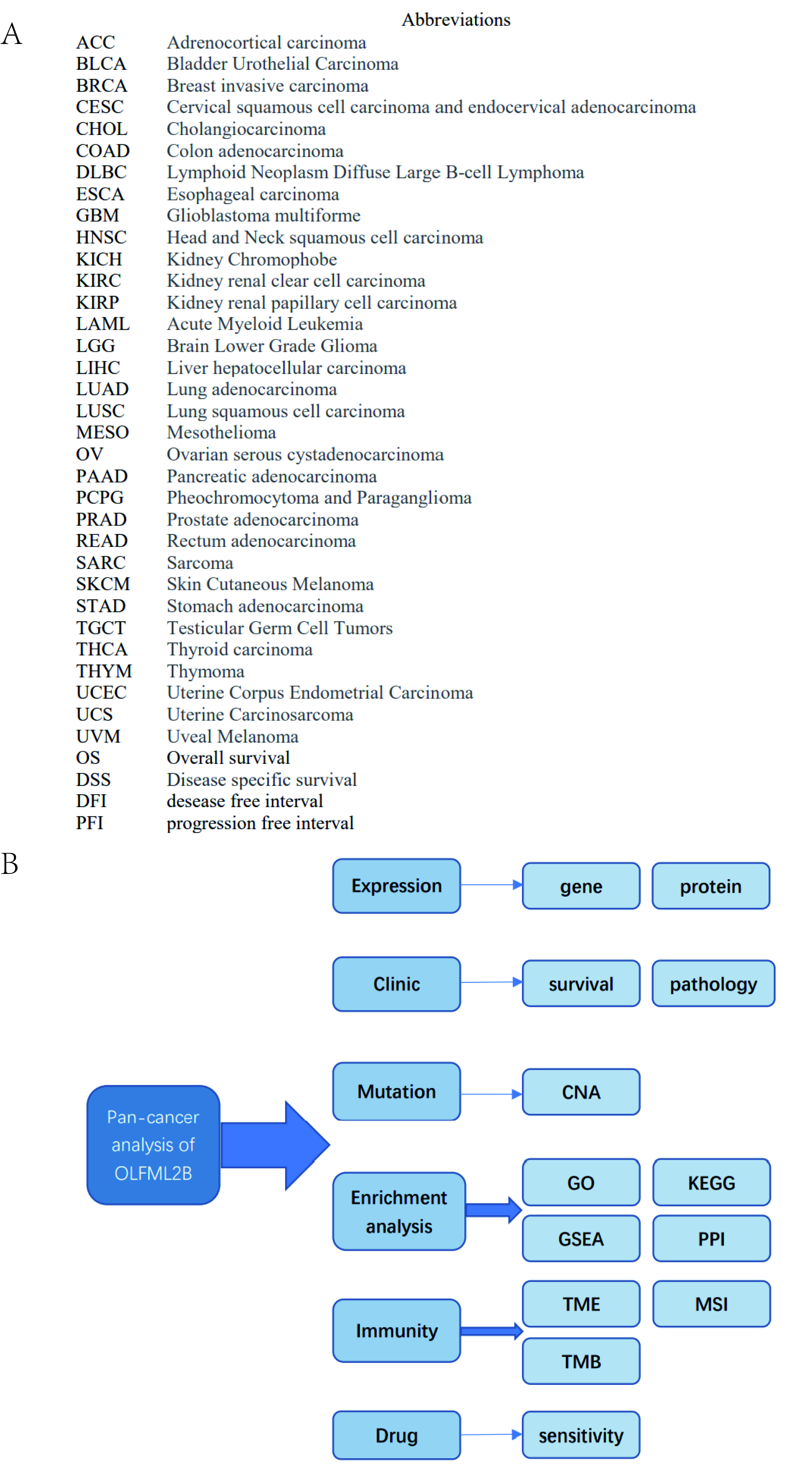

Supplement: Supplementary file 4 [file Image1.TIF]

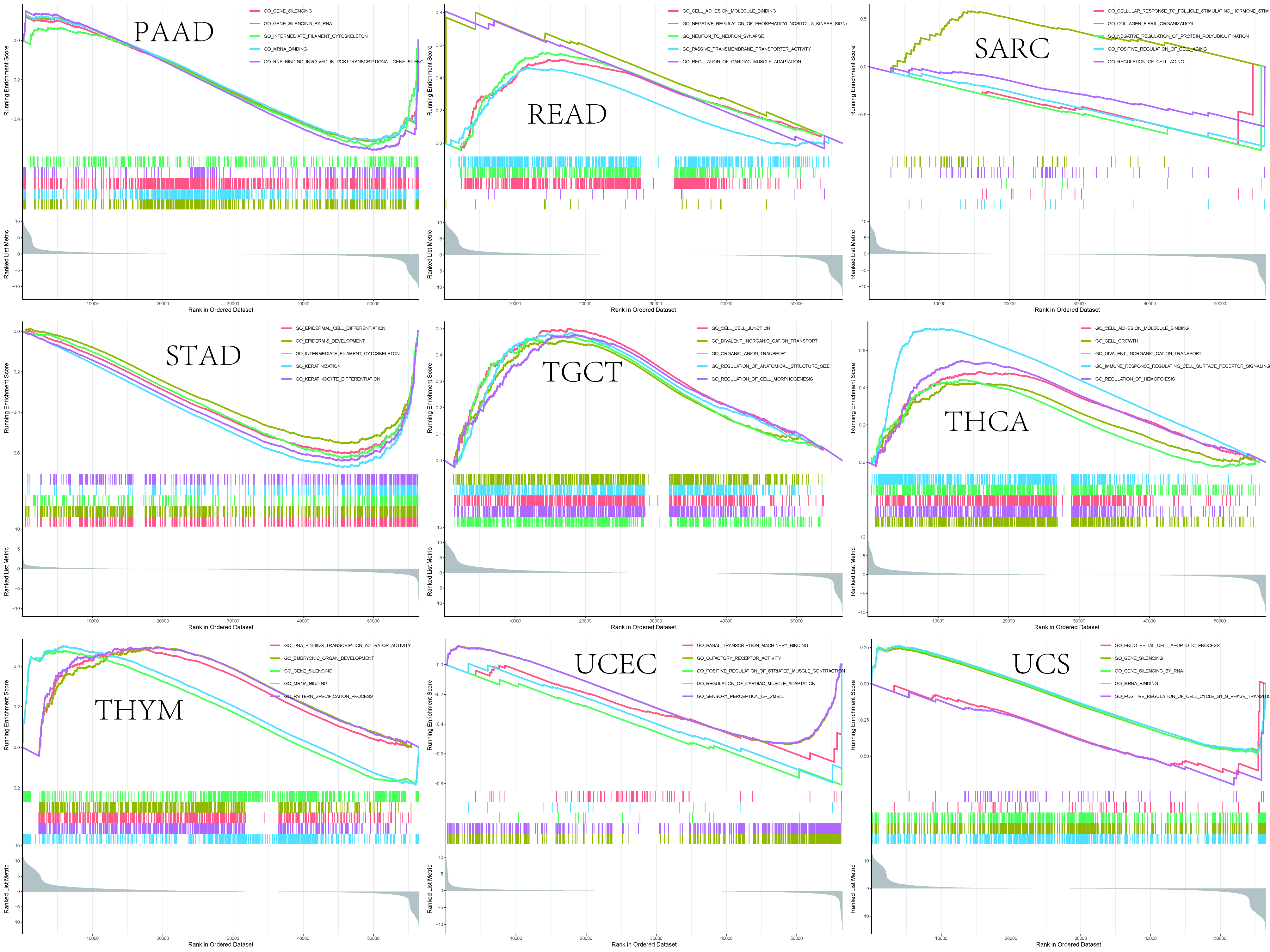

Supplement: Supplementary file 5 [file Image5.TIF]
